# Changes in retinal microvasculature and retinal layer thickness in association with apolipoprotein E genotype in Alzheimer’s disease

**DOI:** 10.1038/s41598-020-80892-z

**Published:** 2021-01-19

**Authors:** Joo Youn Shin, Eun Young Choi, Min Kim, Hyung Keun Lee, Suk Ho Byeon

**Affiliations:** 1 Department of Ophthalmology, Institute of Vision Research, Severance Hospital, Yonsei University College of Medicine, Seoul, Republic of Korea; 2 Department of Ophthalmology, Institute of Vision Research, Gangnam Severance Hospital, Yonsei University College of Medicine, Seoul, Republic of Korea

**Keywords:** Alzheimer's disease, Retinal diseases

## Abstract

Biomarker tests of Alzheimer’s disease (AD) are invasive and expensive. Recent developments in optical coherence tomography (OCT) and OCT angiography (OCTA) have enabled noninvasive, cost-effective characterization of retinal layer vasculature and thickness. Using OCTA and OCT, we characterized retinal microvascular changes in the mild cognitive impairment (MCI) stage of AD and assessed their correlation with structural changes in each retinal neuronal layer. We also evaluated the effect of the *APOE-ε4* genotype on retinal microvasculature and layer thickness. Retinal layer thickness did not differ between MCI patients (40 eyes) and controls (37 eyes, all p > 0.05). MCI patients had lower vessel density (VD) (p = 0.003) of the superficial capillary plexus (SCP) and larger foveal avascular zone area (p = 0.01) of the deep capillary plexus (DCP) than those of controls. VD of the SCP correlated with the ganglion cell layer (r = 0.358, p = 0.03) and inner plexiform layer thickness (r = 0.437, p = 0.007) in MCI patients. *APOE-ε4*-carrying MCI patients had a lower VD of the DCP than non-carriers (p = 0.03). In conclusion, retinal microvasculature was reduced in patients with AD-associated MCI, but retinal thickness was not changed; these changes might be affected by the *APOE* genotype. OCTA of the retinal microvasculature may be useful to detect vascular changes in AD.

## Introduction

Alzheimer’s disease (AD) is a progressive neurodegenerative disease characterized by a gradual decline in memory and cognitive function. The main pathological features are the deposition of Aβ-peptide and hyperphosphorylation of tau protein; however, vascular factors are also considered to be involved in the pathophysiology of AD^1^. Although environmental factors may affect the risk of sporadic AD, studies in twins have suggested that genetic factors play a critical role in late-onset AD^2^. Among the many susceptibility genes for AD, the ε4 allele of apolipoprotein E (*APOE-ε4*) is the major genetic risk factor for both early- and late-onset AD^3^. *APOE-ε4* carriers were reported to have an increased risk of AD (3–15 times) in a gene dose-dependent manner^4^; these carriers showed different treatment responses to amyloid removal therapy and had more vasogenic edema^5^, suggesting a different mechanism of action of *APOE-ε4* in the pathogenesis of AD.

Currently available treatments for AD aim to maximize the remaining activity of the neurons affected by the disease, although they cannot slow down its progression. Thus, there is a great interest in finding biomarkers to screen individuals in the earlier stage of the disease, including those with mild cognitive impairment (MCI) or early AD who would benefit from potential therapy. Current AD biomarker tests mainly include positron emission tomography (PET) or cerebrospinal fluid testing, which is highly invasive and expensive. Noninvasive, technically simple, and inexpensive biomarkers are needed for diagnosis or therapeutic monitoring of AD in the clinical setting or population-wide screening^6^.

The retina shares similar anatomical and physiological features with the brain; numerous studies have reported changes in the retina in patients with AD, suggesting that the retina can be a possible biomarker for diagnosing, screening, and managing AD in clinical practice^7^. The retina can be noninvasively assessed using high-resolution images obtained with optical coherence tomography (OCT). Thinning of the retinal nerve fiber layer (RNFL) and ganglion cell-inner plexiform layer (GC-IPL), and decreased macular volume have been reported to be associated with AD^7,8^. A recent study reported that a thinner RNFL at baseline indicates an increased risk of dementia and AD over the follow-up period, suggesting that OCT measurements of the retina can help identify individuals at a high risk of very early cognitive changes and can help develop better clinical trials^9,10^.

Recent developments in OCT angiography (OCTA) have enabled the characterization of the vasculature in retinal layers at the micrometer level, providing a quantitative assessment of the microvascular structure in the retina. A few studies have reported changes in the microvasculature of AD patients using OCTA, suggesting the presence of retinal microvascular dysfunction^11–15^; however, little is known about the relationship between changes in the retinal microvasculature and retinal neuronal layers and the *APOE-ε4* genotype. In the current study, we aimed to characterize retinal microvascular changes that occur in the MCI stage of AD using OCTA and assess their correlation with retinal structural changes in each retinal neuronal layer. In addition, we evaluated the effect of the *APOE-ε4* genotype on retinal microvasculature and retinal layer thickness.

## Results

### Baseline characteristics and thickness of each retinal layer

A total of 40 eyes from 24 patients with MCI owing to AD and 37 eyes from 31 control subjects were analyzed. All participants were Asians (Korean) and had a global Clinical Dementia Rating (CDR) score of 0.5 and a Mini-Mental State Examination (MMSE) score of > 24. The baseline characteristics of the MCI and control groups are shown in Table 1. There were no statistically significant differences in terms of age (p = 0.15), sex (p = 0.17), spherical equivalent (SE; p = 0.84), visual acuity (p = 0.28), and presence of diabetes mellitus (p = 0.30) and hypertension (p = 0.21) between the two groups. No significant differences were found in total retinal thickness (p = 0.87) and thickness of each retinal layer between the MCI and control groups.

**Table 1:** Demographic characteristics and retinal neuronal layer thickness of the participants.

	MCI (n = 40)	Controls (n = 37)	p-value
Age, mean ± SD, years	72.8 ± 8.6	69.0 ± 10.4	0.15
Sex (M/F), (n/n)	25/15	17/20	0.17
DM (n)	3	6	0.30
HTN (n)	9	14	0.21
OD/OS (n/n)	20/20	19/18	> 0.999
SE, mean ± SD, Diopter	−0.48 ± 2.1	−0.45 ± 1.8	0.84
VA, mean ± SD, logMAR	0.03 ± 0.05	0.04 ± 1.8	0.28
**Retinal neuronal layer thickness, mean ± SD, μm**			
Total retina	315.1 ± 14.2	314.9 ± 14.4	0.87
RNFL	20.0 ± 2.4	19.9 ± 2.5	0.95
GCL	39.1 ± 3.8	38.3 ± 6.3	0.92
IPL	34.6 ± 2.1	33.9 ± 3.6	0.47
INL	37.2 ± 3.9	36.1 ± 3.3	0.20
OPL	33.7 ± 4.7	33.5 ± 5.1	0.83
ONL	67.5 ± 9.9	68.3 ± 8.5	0.96
RPE	15.3 ± 1.7	16.0 ± 1.9	0.25

MCI = mild cognitive impairment, M = male, F = female, DM = diabetes mellitus, HTN = hypertension, SE = spherical equivalent, OD = right eye, OS = left eye, VA = visual acuity, logMAR = logarithm of the minimum angle of resolution, RNFL = retinal nerve fiber layer, GCL = ganglion cell layer, IPL = inner plexiform layer, INL = inner nuclear layer, OPL = outer plexiform layer, ONL = outer nuclear layer, RPE = retinal pigment epithelium, SD = standard deviation.

*p < 0.05.

### Comparison of OCTA parameters between MCI patients and controls

In the superficial capillary plexus (SCP), significantly lower vessel density (VD) was observed in the MCI group than in the control group (p = 0.003), whereas there was no significant difference in the foveal avascular zone (FAZ) area (p = 0.17). In the deep capillary plexus (DCP), there was no difference in VD (p = 0.80) between the MCI and control groups; however, a larger FAZ area was observed in the MCI group than in the control group (p = 0.011) (Table 2).

**Table 2:** Comparison of optical coherence tomography angiography parameters between patients with mild cognitive impairment and controls.

	MCI (n = 40)	Controls (n = 37)	p-value
**SCP, mean ± SD**			
FAZ (mm^2^)	0.31 ± 0.11	0.27 ± 0.09	0.17
VD (%)	14.0 ± 3.9	16.3 ± 2.5	0.003*
**DCP, mean ± SD**			
FAZ (mm^2^)	0.95 ± 0.24	0.80 ± 0.20	0.011*
VD (%)	25.5 ± 1.9	25.6 ± 1.8	0.80

MCI = mild cognitive impairment, SCP = superficial capillary plexus, FAZ = foveal avascular zone, VD = vessel density, DCP = deep capillary plexus, SD = standard deviation.

*p < 0.05.

The comparison of VDs of the SCP in each subfield between the two groups revealed that the MCI group had a significantly lower density than the control group in the whole subfield, except for the nasal inner (p = 0.25) and outer subfields (p = 0.53) (Table 3).

**Table 3:** Comparison of the vessel densities of the superficial capillary plexus in each subfield between the groups.

Subfield, mean ± SD	MCI (n = 40)	Controls (n = 37)	Raw p-value	Benjamini–Hochberg p-value
Central (%)	5.63 ± 3.9	7.8 ± 3.4	0.007*	0.013*
Inner superior (%)	14.2 ± 4.4	16.5 ± 3.3	0.012*	0.018*
Inner nasal (%)	14.1 ± 4.7	15.5 ± 3.8	0.22	0.248
Inner inferior (%)	14.2 ± 4.6	16.1 ± 3.9	0.032*	0.041*
Inner temporal (%)	14.2 ± 4.4	16.8 ± 3.1	0.002*	0.009*
Outer superior (%)	14.4 ± 4.4	17.0 ± 2.4	0.006*	0.013*
Outer nasal (%)	15.9 ± 4.7	16.9 ± 3.4	0.53	0.53
Outer inferior (%)	13.8 ± 4.4	16.5 ± 2.4	0.003*	0.009*
Outer temporal (%)	13.2 ± 4.4	16.7 ± 3.3	< 0.001*	< 0.001*

MCI = mild cognitive impairment, SD = standard deviation.

*p < 0.05.

### Correlation of VD with the thickness of each retinal layer

In the MCI group, the VD of the SCP was significantly correlated with ganglion cell layer (GCL) thickness (p = 0.03) and inner plexiform layer (IPL) thickness (p = 0.007), whereas the VD of the DCP showed no correlation with the thickness of all retinal neuronal layers. Meanwhile, there was no significant correlation between VD and thickness of each retinal layer in both SCP and DCP in the control group (Table 4).

**Table 4:** Correlation of vessel density with each retinal neuronal layer thickness in the two groups.

	MCI		Controls	
	Spearman’s correlation coefficient	p-value	Spearman’s correlation coefficient	p-value
**Correlation with VD of the SCP**				
Total retina	0.224	0.18	0.125	0.52
RNFL	− 0.220	0.19	− 0.122	0.53
GCL	0.358	0.030*	0.120	0.54
IPL	0.437	0.007*	0.170	0.38
INL	0.089	0.60	− 0.073	0.71
OPL	0.133	0.43	− 0.379	0.053
ONL	0.051	0.76	0.335	0.076
RPE	0.141	0.40	0.167	0.39
**Correlation with VD of the DCP**				
Total retina	− 0.101	0.55	0.427	0.20
RNFL	− 0.280	0.09	0.218	0.26
GCL	0.118	0.49	0.095	0.62
IPL	− 0.131	0.44	0.223	0.24
INL	− 0.234	0.16	0.382	0.40
OPL	− 0.118	0.49	0.085	0.66
ONL	0.115	0.50	0.179	0.35
RPE	0.056	0.74	− 0.133	0.49

MCI = mild cognitive impairment, VD = vessel density, SCP = superficial capillary plexus, RNFL = retinal nerve fiber layer, GCL = ganglion cell layer, IPL = inner plexiform layer, INL = inner nuclear layer, OPL = outer plexiform layer, ONL = outer nuclear layer, RPE = retinal pigment epithelium, DCP = deep capillary plexus.

*p < 0.05.

### Comparison of OCT and OCTA parameters between APOE-ε4 carriers and non-carriers

The *APOE* genotype was evaluated in 16 MCI patients, of whom six (10 eyes) were *APOE-ε4* carriers, and 10 (18 eyes) were non-carriers (Table 5). There was no significant difference in terms of age (p = 0.27), sex (p = 0.41), MMSE scores (p = 0.56), and Seoul Neuropsychological Screening Battery (SNSB) scores (p = 0.58). There was no difference in the FAZ area of the SCP (p = 0.65) and the FAZ area of the DCP (p = 0.21) between the two groups. *APOE*-ε4 carriers had lower VD of the DCP than non-carriers (p = 0.03), whereas there was no difference in VD of the SCP (p = 0.83). In terms of retinal layer thickness, no significant difference was observed in each retinal layer with regard to *APOE-ε4* status.

**Table 5:** Optical coherence tomography and optical coherence tomography angiography parameters of *APOE-ε4* carriers and non-carriers.

	*APOE-ε4* +	*APOE-ε4*-	p-value
n	10	18	
Age, mean ± SD, years	71.1 ± 7.1	73.2 ± 6.5	0.27
Sex (M/F), (n/n)	5/5	13/5	0.41
**Retinal microvasculature, mean ± SD**			
FAZ area (mm^2^)-SCP	0.32 ± 0.09	0.31 ± 0.10	0.65
FAZ area (mm^2^)-DCP	1.04 ± 0.24	0.91 ± 0.26	0.21
VD (%)—SCP	14.4 ± 3.8	14.4 ± 3.5	0.83
VD (%)—DCP	24.3 ± 1.8	26.0 ± 2.0	0.03*
**Retinal layer thickness, mean ± SD, μm**			
Total retina	315.8 ± 14.2	316.2 ± 13.8	0.80
RNFL	21.0 ± 3.3	19.8 ± 1.8	0.45
GCL	39.7 ± 4.7	39.7 ± 3.3	0.60
IPL	35.6 ± 1.7	34.6 ± 1.9	0.15
INL	37.9 ± 3.5	36.6 ± 4.2	0.36
OPL	35.2 ± 4.9	32.2 ± 3.1	0.17
ONL	64.7 ± 9.1	69.3 ± 9.5	0.23
RPE	14.7 ± 1.7	15.7 ± 1.4	0.07

APOE = apolipoprotein E, M = male, F = female, FAZ = foveal avascular zone, SCP = superficial capillary plexus, DCP = deep capillary plexus, VD = vessel density, RNFL = retinal nerve fiber layer, GCL = ganglion cell layer, IPL = inner plexiform layer, INL = inner nuclear layer, OPL = outer plexiform layer, ONL = outer nuclear layer, RPE = retinal pigment epithelium, SD = standard deviation.

*p < 0.05.

## Discussion

In the current study, patients with MCI owing to AD had lower VD of the SCP compared with controls who showed no decrease in the thickness of each retinal layer. Furthermore, lower VD was correlated with thinner GCL and IPL in MCI patients, suggesting that changes in the retinal microvasculature occur in the earlier stages of AD, and these changes may precede the reduction of retinal thickness in patients with AD. In addition, we demonstrated differences in the retinal microvasculature under different *APOE-ε4* statuses, indicating that the *APOE* genotype might also affect the changes in retinal microvasculature.

AD is pathologically characterized by amyloid deposits and neurofibrillary tangles. Furthermore, vascular dysfunction has been reported in patients with AD^1^, although it is unclear whether it precedes and contributes to neural death or whether it is an incidental effect of decreased metabolic demand. Cerebral vascular impairment was recognized as one of the earliest pathologic features in AD^16^, and histopathological studies have shown the presence of cerebral capillary degeneration^1,17^. Furthermore, the accumulation of amyloid-beta deposits in the internal vessel walls was suggested to cause the occlusion of vascular structures and decrease the blood flow^13^. The results of recent studies using OCTA show that these vascular changes occur not only in the cerebral vasculature, but also in the retinal microvasculature in patients with AD. Decreased retinal VD has been reported in patients with AD, MCI, and preclinical AD^11–14^. A progressive trend of retinal microvascular loss (both in the SCP and DCP) was observed because of MCI owing to AD, indicating retinal vascular impairment during disease progression, which may contribute to the potential conversion from MCI to AD^11^. Our results also showed lower VD of the SCP and larger FAZ area of the DCP in MCI patients than in controls.

Several studies have reported significant retinal thinning of the GC-IPL and a reduction in macular thickness and macular volume in the eyes of AD patients^7,8^. The proposed hypotheses include retrograde degeneration from loss of cortical neurons or inflammation, amyloid and neurofibrillary tangles disrupting normal retinal cell function, and reduced vascularization. The retinal thickness (peripapillary RNFL and macular GC-IPL) was reported to be generally lower in MCI patients than in controls; however, the magnitude failed to reach a statistical significance in a meta-analysis^7^. The lack of statistical significance may be partly owing to a small number of eligible studies. Moreover, it has been hypothesized that activation of Müller cells and swelling of neurons may occur in the early stages of neurodegeneration, resulting in an increase in macular thickness^18^. In this study, we compared the thickness of all the layers of the retina between MCI patients and controls using automated segmentation software and found no significant differences between the two groups. Our results showed that significantly lower VD was observed in the MCI group than in the control group, although there was no significant difference in the retinal neuronal thickness. This suggests that changes in the retinal microvasculature precede detectable changes in retinal neuronal thickness, and thus, they could be earlier biomarkers.

We also demonstrated a correlation between retinal microvasculature and the thickness of each retinal layer in the eyes of MCI patients. A previous study reported a correlation between loss of retinal microvasculature in the DCP and GC-IPL thinning in the AD group^11^, whereas our results showed that VD of the SCP was correlated with GCL and IPL thickness. Considering that GCL-IPL is mainly supplied by the SCP, our results suggest that these retinal changes, namely GCL and IPL thinning and reduction of retinal microvasculature, are related to each other instead of being independent occurrences. Further exploration of this association is needed to elucidate this relationship, and it may improve our understanding of the dynamics of AD pathology in the nervous system, which has important implications for determining AD risk.

The *APOE-ε4* is the most common genetic risk factor for AD and is linked to other neurodegenerative conditions that affect cognition. *APOE* is known to modulate multiple mechanistic pathways, including cholesterol/lipid homeostasis, synaptic function, glucose metabolism, neurogenesis, tau phosphorylation, neuroinflammation, and aggregation of Aβ in the central nervous system. In addition, *APOE* genotypes differentially modulate the function of the cerebral vasculature, reduce cerebral blood flow, and increase blood–brain barrier leakage and cerebral amyloid angiopathy^19^. Reduced electroretinography responses and lower retinal and choroidal vascular endothelial growth factor were reported in *APOE-ε4* mice^20,21^. In our study, we showed that *APOE-ε4* carriers had decreased VD of the DCP compared with non-carriers, suggesting that the *APOE* genotype causes changes in the microvasculature. Subsequent studies with a larger number of patients are needed.

This study had some limitations. The major limitation was the relatively small sample size, and this may have resulted in a lack of significant findings in some analyses. In particular, since the comparison of OCT and OCTA parameters between APOE***-ε4*** genotypes was an explorative analysis including a small number of eyes, we did not apply multiple testing adjustments. Further investigations with a large sample size are required. We only analyzed a single ethnic group (Korean) thus our results may not be generalizable to other populations. Comparisons among various ethnic groups would be interesting topics for future studies. Although there were no statistically significant differences, older age in the MCI group could have possibly influenced OCTA results. In this study, we analyzed MCI patients who were easily accessible in our clinical practice, but further longitudinal studies including preclinical AD, MCI, and AD participants will be needed to determine whether the evaluation of the retinal microvasculature using OCTA has value as an early biomarker in AD.

In conclusion, retinal microvasculature changes in patients with MCI owing to AD were detected using OCTA, and lower VD of the SCP was correlated with thinner GCL and IPL in these patients. This suggests that changes in the retinal microvasculature precede the reduction in the thickness of the retinal layers in patients with MCI owing to AD. The *APOE* genotype may also affect changes in retinal microvasculature. Evaluation of the retinal microvasculature may be used as a potential biomarker to detect vascular changes in MCI owing to AD and could be a new imaging target for early diagnosis and management of AD.

## Methods

This case–control study was approved by the institutional review board of Yonsei University College of Medicine (IRB approval number: 3–2018-0156) and was conducted in accordance with the tenets of the Declaration of Helsinki. All study participants provided informed consent. We recruited patients and controls from the Gangnam Severance Hospital between September 2017 and December 2018. Trained and qualified neurologists made the diagnosis of MCI owing to AD based on the National Institute on Aging-Alzheimer’s Association criteria^22^. All MCI subjects tested positive for Aβ deposition in the brain on^18^F‐florbetaben amyloid PET; brain magnetic resonance imaging and laboratory tests (e.g., thyroid function tests, serum vitamin B12, and folate levels) were performed to exclude other causes of cognitive decline. The MCI group underwent a neuropsychological test battery (MMSE, CDR, and SNSB^23^) for the assessment of global cognitive function. Genetic testing for *APOE* was performed only in patients who agreed to undergo the testing. The control group comprised of volunteers who were scheduled to undergo cataract surgery and who were clinically assessed as cognitively normal based on clinical interviews with patients and their caregivers. The control group subjects had no history of amnesia and no previous history of head trauma or neurological or psychiatric illness. We excluded subjects previously diagnosed with clinically apparent AD, uncontrolled hypertension, or uncontrolled diabetes.

All subjects underwent corrected distance visual acuity (logMAR), intraocular pressure (mmHg), and spherical equivalent measurements. Detailed anterior segment and fundus examinations were performed, and wide-field color fundus images were taken using a laser scanning ophthalmoscopy device (Optomap; Optos Plc., Dunfermline, UK). Examination of the microvasculature and retinal thickness of each layer of the macula was performed using two different devices, ZEISS OCTA and SPECTRALIS OCT, respectively, on the same day.

We performed OCTA (ZEISS HD-OCT Model 5000 instrument with AngioPlex, Carl Zeiss Meditec, Dublin, CA, USA) to examine the retinal microvasculature covering a macular area of 6 × 6 mm centered on the fovea for the SCP and DCP of the retina. The projection artifacts of the superficial layer were removed in the deep-layer images using built-in software. All images were exported into the Image J 1.50 software (National Institutes of Health, Bethesda, MD, USA) to measure the FAZ area and VD. We manually outlined the FAZ using the polygon selection tool^24,25^ and calculated the VD of the 6 × 6-mm macula, except the FAZ (central foveal 0.5-mm radius area), by image thresholding, binarization, and skeletonization according to the methods described in a previous study^26^. Two independent researchers (EYC and JYS) obtained and evaluated OCTA findings, and the average values were used for the statistical analysis. In the analysis of the FAZ and VD in Image J, and manual edits of OCTA segmentation, researchers were masked to participant characteristics to avoid bias.

To examine the thickness of each retinal layer, we used SPECTRALIS OCT (Spectralis HRA + OCT; Heidelberg Engineering, Franklin, MA, USA) to scan a macular area of 6 × 4 mm centered on the fovea. The retinal segmentation software accompanying the device was used to identify each retinal neuronal layer and quantify its thickness; the software automatically calculated the average retinal thickness of each retinal layer. For the analysis, retinal layers were divided into RNFL, GCL, IPL, inner nuclear layer, outer plexiform layer, outer nuclear layer, and retinal pigment epithelium (RPE).

We reviewed all segmentation images of OCTA and OCT and manually modified significant segmentation errors. We excluded eyes with retinal diseases (e.g., age-related macular degeneration, diabetic retinopathy, epiretinal membrane, and macular hole), optic nerve diseases (e.g., glaucoma, and ischemic optic neuropathy), significant media opacity with poor quality (signal strength < 70 on OCTA and < 25 on OCT), or high refractive error over ± 6D from the analysis. Thus, eight eyes from the MCI group (two eyes with epiretinal membrane, five eyes with drusen or RPE changes, one eye with low signal strength) and 25 eyes of controls (three eyes with epiretinal membrane, seven eyes with drusen or RPE changes, 15 eyes with low signal strength) were excluded from the analysis.

When analyzing OCT and OCTA images, we used the average values of the standard retinal subfields, namely central, superior, temporal, inferior, and nasal quadrants of the inner and outer rings as defined in the Early Treatment Diabetic Retinopathy Study^27^. The diameters of the central, inner, and outer rings were 1, 3, and 6 mm, respectively.

### Statistical analysis

For comparison of the baseline characteristics, the thickness of each retinal layer, and OCT parameters between the MCI group and controls, we used the Mann–Whitney test for continuous variables and Fisher’s exact test for categorical variables. In the comparison of the VD of the SCP in each subfield between the MCI and controls groups, the Benjamini–Hochberg method was used for multiple testing adjustment. To assess the relationship between VD and retinal layer thickness, Spearman’s correlation was used. Because the covered macular area differed in the two devices (6 × 6 mm in OCTA and 6 × 4 mm in OCT), we used the average values of VD and retinal layer thickness in the inner ring in the analysis. We categorized the *APOE* genotype into *APOE-ε4* carriers (10 eyes from 6 patients) and non-carriers (18 eyes from 10 patients) because of the low prevalence of *APOE-ε4* homozygotes. We compared OCT and OCTA parameters between *APOE-ε4* carriers and non-carriers using the Mann–Whitney test for continuous variables and Fisher’s exact test for categorical variables. Statistical analysis was performed using SPSS software 21 (SPSS, Inc., Chicago, IL, USA), and p-values < 0.05 were considered statistically significant.
